# Death due to Cardiac Arrest in a Young Female With Highly Suspected COVID-19: A Case Report

**DOI:** 10.7759/cureus.10127

**Published:** 2020-08-30

**Authors:** Sherif Mohamed, Osama Abo El-Hassan, Magda Rizk, Jumana H Ismail, Aml Baioumy

**Affiliations:** 1 Chest Diseases and Tuberculosis, Faculty of Medicine, Assiut University, Assiut, EGY; 2 Pulmonary Medicine, Faculty of Medicine, Cairo University, Cairo, EGY; 3 Anaesthesiology, Faculty of Medicine, Cairo University, Cairo, EGY; 4 Chest Diseases, Faculty of Medicine, Cairo University, Cairo, EGY

**Keywords:** covid-19, cardiac arrest, mortality, young, female, false-negative, pulmonary embolism, coagulopathy, case report

## Abstract

Despite the common clinical presentations of the pandemic coronavirus disease of 2019 (COVID-19) being well-known, there remain issues on its atypical or rare presentations. Moreover, despite the known risk factors for severe COVID-19 are cardiovascular disease, diabetes mellitus, hypertension, chronic lung disease, and advanced age, still younger patients suffer from this disease. Herein, we present a case report of a 28-year-old female patient who was presented to the ED with cardiac arrest, then died within 12 hours. First swab testing by reverse transcription-polymerase chain reaction (RT-PCR) came negative. However, she has typical CT features of COVID-19 pneumonia, along with an echocardiographic picture of acute cor pulmonale. Though it is rare, cardiac arrest can happen in young apparently healthy patients with COVID-19. As COVID-19 patients are commonly having clotting disorders, endothelial and organ dysfunction, coagulopathy, and liable for pulmonary thromboembolism (PTE), it is important to select those COVID-19 patients who are at higher risk of PTE, and practice CT pulmonary angiography (CTPA) for the diagnosis of PTE, especially in case of significant increase of D-dimer values.

## Introduction

Despite the fact that common classical clinical presentations of the pandemic coronavirus disease of 2019 (COVID-19) are well-known, there remain issues on its atypical or rare presentations. In a report to the United States Centers for Disease Control and Prevention (CDC) of over 370,000 confirmed COVID-19 cases [1], the reported symptoms in descending order were as follows; cough (50%), fever (subjective or >100.4°F/38°C; 43%), myalgia (36%), headache (34%), and dyspnea (29%). Less common symptoms included sore throat (20%), diarrhea (19%), nausea and/or vomiting (12%), and loss of smell or taste, abdominal pain, and rhinorrhea (<10%) [1].

Severe COVID-19 can occur in otherwise healthy individuals of any age, but it predominantly occurs in adults with advanced age or underlying medical comorbidities [2]. Recently, the CDC has created a list of certain comorbidities that have been associated with severe disease (defined as infection resulting in hospitalization, admission to the ICU, intubation or mechanical ventilation, or death) [3]. Established risk factors include: cancer, chronic kidney disease, chronic obstructive pulmonary disease (COPD), immunocompromised state from a solid organ transplant, obesity, serious cardiovascular disease, heart failure, coronary artery disease, cardiomyopathies, sickle cell disease, and type 2 diabetes mellitus. 

Herein, we present a case report of a 28-year-old female patient who presented to the ED with cardiac arrest and then died within 12 hours. Swab testing by reverse transcription-polymerase chain reaction (RT-PCR) came negative on the next day. However, she had typical CT features of COVID-19 pneumonia. This atypical and severe presentation of COVID-19 in a young patient could have significant impacts on diagnostic and therapeutic strategies of such patients. 

## Case presentation

**Figure 1:**
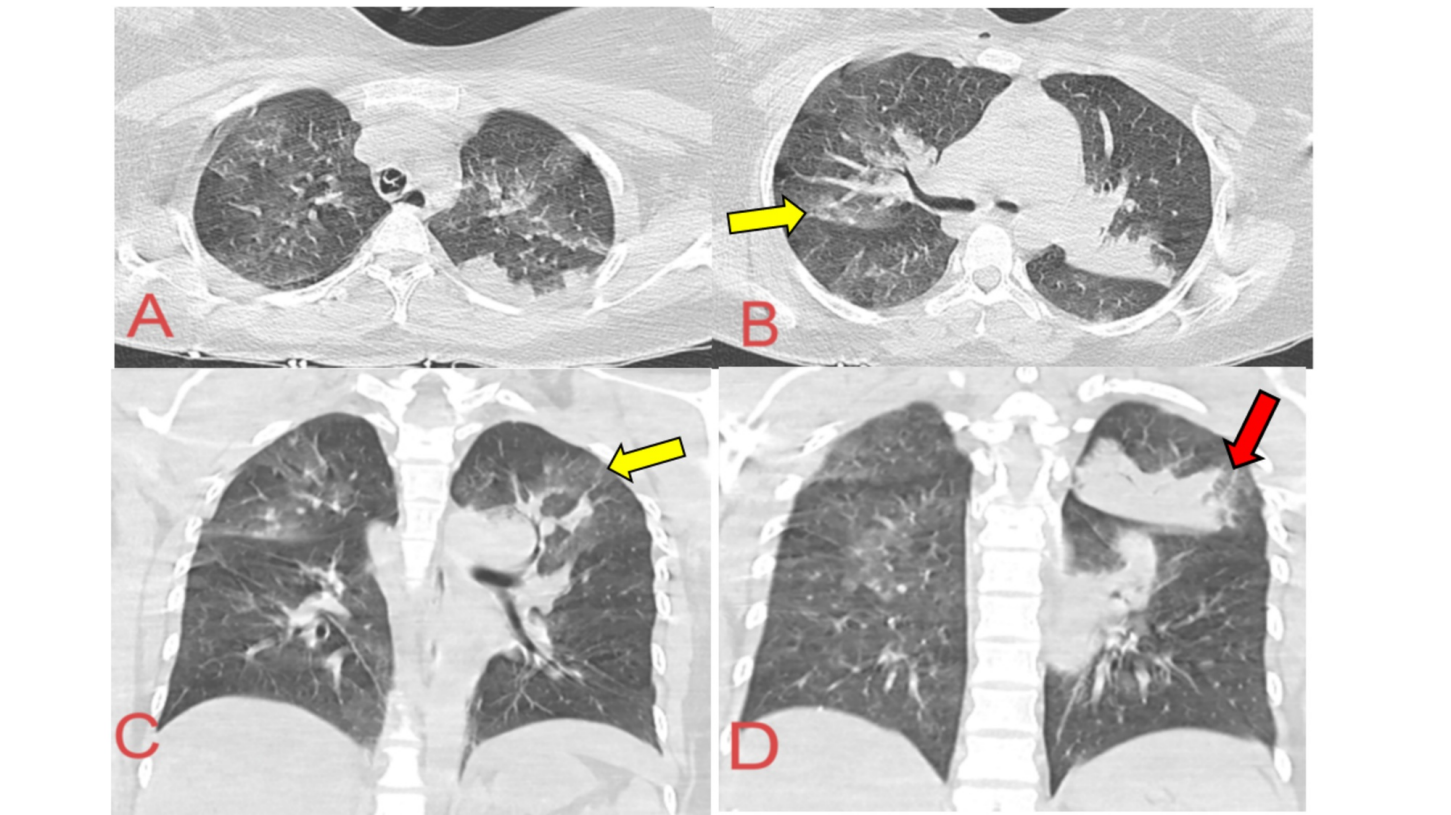
CT chest images of cardiac arrest in a young female patient. A&B: Axial views showing bilateral mosaic changes and peripheral ground glass opacities (GGOs) in both upper lobes (yellow arrows) with consolidation of the left upper lobe (red arrow). C&D: Coronal views confirming the same findings.

A 28-year-old Egyptian female patient was brought by her husband to the ED in a state of cardiac arrest. Cardio-pulmonary resuscitation (CPR) was immediately started and she revived after seven minutes. She was intubated and mechanically ventilated. History was taken from the husband that the patient is a nonsmoker, with two days history of cough and mild shortness of breath, with no fever. Two hours before arrest she felt marked shortness of breath, chest tightness, then developed fainting attack with marked pallor and cold extremities. No history of close contact to COVID-19 suspected or confirmed case in the last two weeks. No history of convulsions, vomiting, headache, or gastrointestinal symptoms. No past history of any chronic medical or pulmonary illnesses. After resuscitation, baseline clinical examination of the chest, heart abdomen was unremarkable, except for tachycardia. No clinical signs were suggestive of deep venous thrombosis (DVT) of the lower limbs. As the patient's symptoms were mainly respiratory -- at the time of COVID-19 pandemic -- the resuscitating team decided to do urgent plain CT of the brain and chest before transferring the patient to the ICU. CT brain was unremarkable, whereas surprisingly CT chest revealed extensive bilateral wide-spread, more peripherally situated parenchymal ground glass opacities (GGOs) and consolidations in almost all lobes of both lungs (Figure 1).

**Figure 2:**
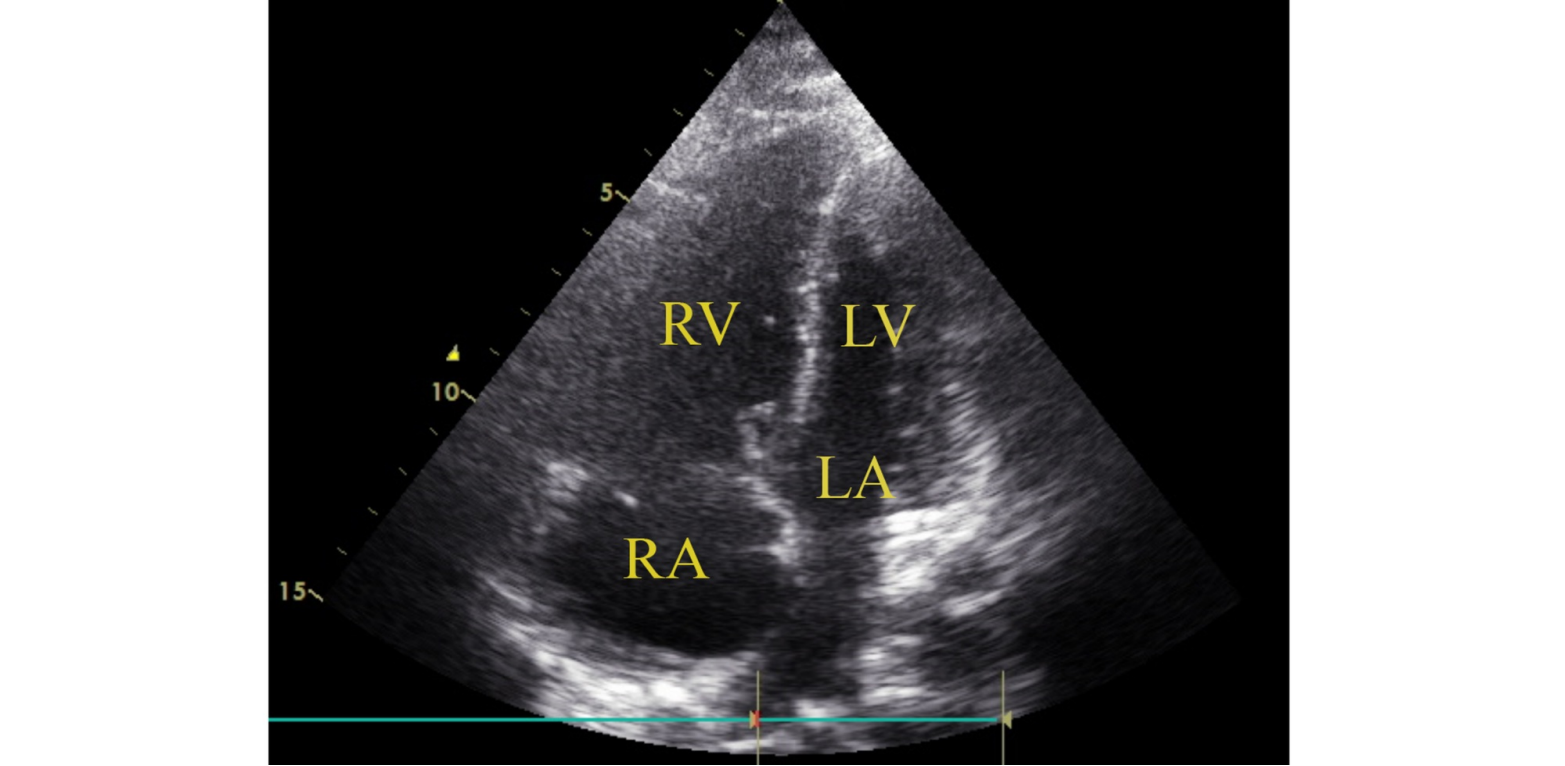
Echocardiography shows acute cor pulmonale. Hugely dilated right side of the heart. Dilated right ventricular dimension, with reverted Brenheimic effect and paradoxical septal motion. Normal left ventricular chamber internal dimensions with good systolic function. No regional resting wall motion abnormalities. RV, right ventricle; LV, left ventricle; RA, right atrium; LA, left atrium

At the ICU, the patient was on mechanical ventilation, FIO2 of 100%, with vital signs of blood pressure 70/40 mmHg, temperature 35.5°C, respiratory rate of 35 cycles/minute, and O2 saturation of 88%. The treating and ICU teams decided ventilator strategy for acute respiratory distress syndrome (ARDS), prone positioning, IV vasopressors, septic workup, coagulation profile, and to do tracheal secretions swabbing for RT-PCR. Her laboratory workup revealed normal total white blood count (WBC) with relative neutropenia and lymphocytosis, hemoglobin (Hb) level of 11.2 g/dL, and normal platelet count. She had normal renal functions and electrolytes with double fold rising of liver enzymes. D-Dimer was 6.1 ug/mL. Serum testing for pregnancy (B-HCG) came negative. Electrocardiography (ECG) revealed right ventricular strain and right bundle branch block (RBBB). Bedside 2D echocardiography was carried out and revealed a picture of acute cor pulmonale (Figure 2).

Ejection fraction (EF) was 64%. Despite all measures, the patient remained hypoxic and hypotensive for 12 hours after intubation. Then, she had a second cardiac arrest, and CPR was initiated; however, she did not revive and was declared dead after 12 hours from her intubation. Next day, the result of RT-PCR came negative for SARS-CoV-2. A consent was obtained from the patient husband for publication of this case report.

## Discussion

Herein, we present a case report of a 28-year-old female who presented with cardiac arrest, with typical CT findings of COVID-19 pneumonia, then died within 12 hours. This atypical and severe presentation for COVID-19 is strange and uncommon for many reasons: (1) young age; (2) female; (3) apparently healthy with apparently good underlying cardiopulmonary reserve and no underlying risk factors of COVID-19 or its severe form; (4) rapid deterioration in few hours, up to death; and (5) first sample was negative for SARS-CoV-2 by RT-PCR. Unfortunately, there was no time for a second confirmatory sample.

Our current knowledge for clinical presentation of COVID-19 is that 81% of infected individuals have mild symptoms, 14% have severe symptoms requiring hospitalization, while 5% become critically ill requiring mechanical ventilation. These differences in response are likely the result of degree of viral load, host immune response, age of the patient, and presence of co-morbidities [4]. However, because the obligate receptor for the virus spike protein, human angiotensin converting enzyme (ACE-2) is expressed in epithelial cells throughout the body, including the lungs, heart, kidney, and even the endothelium [5]; it is not surprising that 8%-28% of patients with COVID-19 infections have evidence of cardiac injury with elevated troponin [6]. Some patients with COVID-19 infections die from cardiac arrest, likely as a result of a combination of primary cardiac involvement, or manifestation of systemic involvement such as severe hypoxia, multi-organ dysfunction syndrome, or systemic inflammatory response syndrome [4]. Acute pulmonary embolism (PE), reported in COVID-19 patients, has been shown to be a cause of clinical deterioration in viral types of pneumonia, as well [7].

Patients with COVID-19 often show clotting disorders, with organ dysfunction and coagulopathy, resulting in higher mortality [8]. Critical data came from the analysis of coagulation tests in samples collected on admission and during the hospital stay of COVID-19 patients. Nonsurvivors had significantly higher D-dimer and fibrinogen-degradation product (FDP) levels, and longer prothrombin time (PT) vs. survivors on admission [9]. Moreover, the clinical diagnosis of disseminated intravascular coagulation (DIC) was observed in nonsurvivors during late stages of hospitalization [9]. Moreover, endothelial dysfunction is a key determinant in hypertension, thrombosis, and DIC, which is common in patients with severe COVID-19 [10].

Back to the clinical presentation of our case report, we thought that the patient had the diagnosis of severe COVID-19 disease, despite that swab came negative. International guidelines have reported that a considerable number of patients could have false negative testing for SARS-CoV-2, depending on the type of collected specimen [11]. Supporting this observation, recent studies had shown the diagnostic significance of CT chest in diagnosing COVID-19 patients who had initial negative testing result(s) by RT-PCR [12]. Despite that the cause of death in this patient is not clear, we think that it is attributed to massive PE. This possibility is supported by the bedside echocardiographic findings and high D-Dimer levels. Unfortunately, time was not enough to carry out CT pulmonary angiography (CTPA) to confirm this diagnosis. Recently, autopsy studies performed for COVID-19 patients were shown. In patients who died from COVID-19-associated respiratory failure, the histologic pattern in the peripheral lung was diffuse alveolar damage with perivascular T-cell infiltration. The lungs also showed distinctive vascular features, consisting of severe endothelial injury associated with the presence of intracellular virus and disrupted cell membranes. Histologic analysis of pulmonary vessels showed widespread thrombosis with microangiopathy [13].

Unfortunately, the patient’s husband did not agree for post-mortem examination of our patient. Lessons that could be learned from this case is that, it is important to select COVID-19 patients at higher risk of PE, and practice CTPA for the diagnosis of pulmonary thromboembolism (PTE) especially in case of significant increase of D-dimer values. Anticoagulation could be a necessary therapy to control and reduce pro-thrombotic events, as well as to prevent PE, in patients with COVID-19 [9].

## Conclusions

Despite it is rare, cardiac arrest could happen in young apparently healthy patients with COVID-19. The clinicians dealing with suspected or confirmed COVID-19 cases should always be alert. As COVID-19 patients are commonly having clotting disorders, endothelial and organ dysfunction, coagulopathy, and liable for PTE. We recommend that it is of crucial importance to select those COVID-19 patients at higher risk of PTE and practice CT pulmonary angiography for the diagnosis of PTE, especially in case of significant increase of D-dimer values. If no clear contraindication(s), anticoagulation should be started early to control and reduce pro-thrombotic events in patients with COVID-19.
